# Cerebrospinal fluid CEFA composition is enriched in saturated fatty acids and it is altered in Alzheimer’s disease

**DOI:** 10.1016/j.jlr.2026.101034

**Published:** 2026-04-03

**Authors:** Chiara Pavanello, Alice Ossoli, Chiara Comi, Marta Turri, Lucio Tremolizzo, Elisa Conti, Paolo D'Incecco, Alberto Barbiroli, Paolo Parini, Nico Mitro, Donatella Caruso, Giulia Sierri, Francesca Re, Clizia Chinello, Claudia Fumagalli, Fulvio Magni, Karl Fernandes, Laura Calabresi

**Affiliations:** 1 Centro E. Grossi Paoletti, Dipartimento di Scienze Farmacologiche e Biomolecolari “Rodolfo Paoletti”, Università degli Studi di Milano, Milano, Italy; 2 Research Centre on Aging, CIUSSS de l'Estrie-CHUS, Department of Medicine, University of Sherbrooke, Sherbrooke, Canada; 3 Neurology Unit, IRCCS “San Gerardo dei Tintori”, Monza, Italy; 4 School of Medicine and Surgery, University of Milano-Bicocca, Milano, Italy; 5 Department of Food, Environmental and Nutritional Sciences (DeFENS), Università degli Studi di Milano, Milano, Italy; 6 Karolinska Institute, Stockholm, Sweden; 7 Dipartimento di Scienze Farmacologiche e Biomolecolari “Rodolfo Paoletti”, Universitàdegli Studi di Milano, Milano, Italy; 8 Department of Experimental Oncology, European Institute of Oncology (IEO) IRCCS, Milano, Italy; 9 Proteomics and Metabolomics Unit, Department of Medicine and Surgery, University of Milano-Bicocca, Milano, Italy

**Keywords:** lecithin:cholesterol acyltransferase, LCAT, cholesterol esterification, fatty acids

## Abstract

Cholesterol esterification is a fundamental step in cholesterol metabolism and transport, and in humans, it is operated by three enzymes. Lecithin:cholesterol acyltransferase (LCAT) is responsible of cholesterol esterification in plasma and other biological fluids including cerebrospinal fluid (CSF), where it is mainly activated by apolipoprotein E. Esterification of cholesterol within cells is instead operated by sterol O-1 and O-2 acyltransferases (SOAT1 and SOAT2). SOAT1 is expressed in all cell types, while SOAT2 is expressed in hepatocytes and enterocytes, where it produces cholesteryl esters (CEs) to be assembled within VLDL and chylomicrons. LCAT and SOAT1/2 have different substrate specificity; LCAT has a preference for the unsaturated fatty acids, while the SOAT enzymes prefer the saturated (SFAs) and monounsaturated fatty acids (MUFAs). Here, we show that CSF CEs have a different composition compared to plasma CEs and specifically are more enriched in SFAs and MUFAs, typical substrates of the SOAT2 enzyme, and less frequently used by LCAT. Protein and RNA analysis in astrocytes, the main lipoprotein-producing cells in the central nervous system, excluded the presence of SOAT2, thus suggesting that CSF CEs are products of the LCAT enzyme. In line with this hypothesis, CSF phosphatidylcholine, the substrate of LCAT, is enriched in SFAs and MUFAs and depleted in polyunsaturated fatty acids. Moreover, we show that in Alzheimer's disease patients, CSF CEs are enriched in SFA, thus adding new insights into our recent observation that LCAT-mediated cholesterol esterification is hampered in Alzheimer's disease. In conclusion, the present findings not only clarify the enzymatic origin of CSF CEs but also open avenues for developing enzyme-specific biomarkers and therapeutic strategies aimed at restoring lipid homeostasis in the brain.

Cholesterol esterification is a fundamental step in cholesterol metabolism and transport, and in plasma around 70% of cholesterol is present in the esterified form. The enzyme responsible of cholesterol esterification in plasma and other biological fluids is lecithin:cholesterol acyltransferase (LCAT), which is produced by the liver and circulates bound to plasma lipoproteins (1). ApoA-I is the major LCAT activator in plasma, and the unesterified cholesterol carried by the small discoidal nascent HDLs represent the best LCAT substrate (1). LCAT is also synthesized in the central nervous system (CNS), mainly by glial cells, and it esterifies cholesterol in cerebrospinal fluid (CSF) (2). In CSF, LCAT is mainly activated by apoE, which is produced by brain cells (2). ApoA-I is not produced within the CNS, but it is present in the CSF in amount similar to apoE (2); this apoA-I is plasma derived and it is a part of small discoidal HDL, which can cross the blood-brain barrier (2), and could contribute to LCAT activation.

Two intracellular enzymes operate the esterification of cholesterol within the cells: sterol O-1 acyltransferase (SOAT1), expressed in all cell types, and sterol O-2 acyltransferase (SOAT2), which is mainly expressed in hepatocytes and enterocytes (3), where it produces cholesteryl esters (CEs) to be assembled within VLDL and chylomicrons (4). The *SOAT1* gene encodes a 550 amino acid protein, while the *SOAT2* gene encodes a protein of 522 amino acids. Human SOAT1/2 have ∼50% protein sequence identity (5). In humans, a small portion of the circulating CEs originates from SOAT2, as shown by the very small amounts of CEs in plasma of carriers of complete LCAT deficiency (∼8% of controls) (6). The potential contribution of SOAT2 to brain lipoproteins has never been investigated.

We have recently set techniques to analyze cholesterol esterification in CSF and showed that CSF cholesterol is mainly present in the esterified form, although at a lesser extent than in plasma (60% *versus* 75%) (7). In the present proof-of-concept study, we extended the analyses by investigating the fatty acid composition of CSF CEs, thus defining their enzymatic origin. In addition, we extended our recent results showing that the esterification process is hampered in the CSF of patients with Alzheimer's disease (AD) (7) by analyzing the fatty acid composition of CEs in CSF of AD patients.

## Materials and Methods

### Subjects

Cognitively normal (CN) controls and AD patients were recruited at the IRCCS “San Gerardo dei Tintori” within a large trial (7). Twelve AD patients and four age- and sex-comparable CN controls (AD:controls ratio, 3:1) have been recruited for the present study. CN controls were without personal or family history of neurological or psychiatric disorders, and absence of cognitive impairment was established by a clinical interview, including a Mini-Mental State Examination score >26, Clinical Dementia Rating = 0, and by CSF AD-biomarker profile. AD patients were evaluated for clinical manifestations of cognitive symptoms and AD was diagnosed according to the NINCDS-ADRDA criteria. Alternative diagnoses were excluded by the evaluation of CSF biomarkers, brain magnetic resonance imaging scan, and a routine extensive neuropsychological test battery. Patients that did not display A+T+N+ profile were excluded. Patients and controls with recent infections or surgery (6 months), or under anti-inflammatory, corticosteroid, or immunosuppressive drug treatments, or affected by kidney or liver failure were excluded. The study was conducted in accordance with the guidelines of the Declaration of Helsinki and its later amendments and was approved by the internal ethical committee (Comitato Etico Brianza, approval #3267 of May 21, 2020) and all subjects signed an informed consent.

To analyze *APOE* genotype, total genomic DNA was isolated from peripheral blood using a commercial DNA extraction kit (Qiagen, Venlo, The Netherlands). DNA amplification was subsequently carried out with specific primers (7).

### Plasma and CSF sample collection and preparation

Blood samples were collected from patients and controls after overnight fasting and centrifuged immediately. Plasma aliquots were frozen at −80°C and slowly defrosted at the time of use.

CSF was collected by lumbar puncture using a 21-gauge needle in 10-ml polypropylene tubes. Aliquot of the CSF was used for routine analysis including leukocyte count, erythrocyte count, glucose concentration, and total protein concentration. The remaining CSF was centrifuged at 2000 *g* for 10 min at room temperature to eliminate cells and transferred to new polypropylene tubes and stored at −80°C until biomarker analysis. Aβ1-40, Aβ1-42, T-tau, and P-tau were evaluated using commercially available kits Fujirebio© using the Lumipulse G600II instrument. Cut-off values for AD diagnosis were the following (normal values are reported): Aβ1-42 > 599 ρg/ml; Aβ1-40 n.a.; T-tau < 404 ρg/ml; P-tau < 56.5 ρg/ml; Aβ1-42/40 ratio > 0.069; Aβ1-42/T-tau ratio > 1.275; and Aβ1-42/P-tau ratio > 8.1.

### Plasma and CSF biochemical analyses

A complete lipid-lipoprotein profile, including total cholesterol, HDL-cholesterol, triglycerides (TGs), apoA-I, apoA-II, apoE, and apoB, was determined using a Roche Integra c311 autoanalyzer. LDL-cholesterol was calculated by the Friedewald's formula. Plasma unesterified cholesterol and phospholipids were determined by enzymatic techniques using external calibration curves generated with commercial standard solutions (DiaSys Diagnostic Systems, Holzheim, Germany). Unesterified cholesterol was measured using a cholesterol oxidase and peroxidase-based reaction, with absorbance read at 510 nm. Phospholipid levels were determined after phospholipase D hydrolysis followed by choline oxidase and peroxidase reactions, with absorbance measured at 505 nm. Concentrations were calculated from the respective standard curves (7).

The amount of CEs was calculated by subtracting unesterified cholesterol from total cholesterol and the difference was multiplied by 1.68 to have a precise estimation of the CE mass.

CSF total and unesterified cholesterol were measured by HPLC and phospholipids were measured with a commercial kit as described in (7). ApoE and apoA-I were determined in CSF by SDS electrophoresis followed by immunodetection with specific antibodies. Calibration curves were generated using purified human apoA-I (isolated from healthy donors) and recombinant human apoE (Sigma-Aldrich, St. Louis, MO) standards at increasing concentrations within the linear detection range. CSF samples were separated by SDS-PAGE, transferred to membranes, and recognized with specific primary antibodies against apoA-I (1:1000, Sigma-Aldrich) and apoE (1:2000, Sigma-Aldrich). Protein bands were visualized by chemiluminescence and quantified by densitometric analysis. Apolipoprotein concentrations were calculated by interpolation from the respective standard curves (7). To exclude blood contamination, all CSF samples were tested for apoB, which is not normally found in CNS.

The cholesterol esterification process was evaluated in plasma samples by measuring the cholesterol esterification rate (CER), reflecting the ability of endogenous LCAT to esterify cholesterol in endogenous lipoproteins, and LCAT activity, reflecting the enzyme's ability to esterify cholesterol in exogenous reconstituted HDL (8). Cholesterol esterification in CSF was evaluated using the plasma CER method, with minor modifications to account for the very low concentrations of substrate and enzyme in CSF. Briefly, CSF samples were incubated for 6 h (instead of 1 h as for plasma), and unesterified cholesterol was quantified before and after incubation by HPLC. The decrease in unesterified cholesterol was then used to calculate CSF CER, expressed as nmol CE/ml/h.

### Plasma and CSF lipidomic analysis

For CEs analysis, 1 μl of methyl ter-butyl ether was added to 100 μl of plasma or CSF samples, the supernatant organic phase was extracted and resuspended with a solution of 2-propanol, acetonitrile, formic acid, and ammonium acetate (Mobile Phase B, MPB. Plasma samples were diluted in MPB 1:200 (v:v), while CSF samples were diluted 1:4 (v:v) before injection in triplicate. Samples were analyzed for the positive ion modes using Dionex Ultimate 3000 nano-LC system (Sunnyvale CA), with a micro flow selector, connected to Orbitrap Exploris™ 240 Mass Spectrometer (Thermo Fisher Scientific) equipped with heated electrospray ionization. Chromatographic separation was achieved on an Acclaim PepMap® RSLC (Thermo Fisher Scientific™), 150 mm (length) × 300 μm (ID) × 3 μm (particle size) using mobile phase A (0.1% formic acid and 10 mM ammonium acetate in water/acetonitrile (60/40)) and MPB (0.1% formic acid and 10 mM ammonium acetate in 2-propanol/acetonitrile (90/10)) at a flow rate of 8 μl/min. The column and autosampler temperatures were set at 45°C and 7°C, respectively. The sample injection volume was 1 μl. Stop run MS spectra were collected over an m/z range of 250–800 Da at 120,000 resolutions, operating in the data-dependent mode, 5 scans. Polarity: positive. Higher-energy collisional dissociation energy (%) was set at 40 for all analytes, except for cholesterol that was 80. Data processing was carried out using the software Compound Discoverer™ (ver. 3.3; Thermo Fisher Scientific).

For phospholipid composition analysis, internal standards solution (IS: Splash Lipidomix Internal Standards, Avanti Polar) and dichloromethane:methanol solution (2:1, v:v) were added to the CSF. The supernatant organic phase was collected and the samples were diluted with 25 μl a solution made of 2-propanol:acetonitrile (90:10, v:v), 0.1% formic acid and 10 mM ammonium acetate. All samples have been analyzed for the positive ion modes using ExionLC™ AD system (SCIEX) connected ZenoTOF 7,600 System (SCIEX) equipped with Turbo V™ Ion Source with ESI Probe. The chromatographic separation on a Kinetex® EVO (Phenomenex®) 100 (length) x 2.1 mm (ID) × 1.7 μm (particle size) was achieved using, as mobile phase A, water:acetonitrile (60:40, v:v) and, as MPB, 2-propanol:acetonitrile (90:10, v:v), both containing 10 mM ammonium acetate and 0.1% of formic acid. The flow rate was 0.400 ml/min and the column temperature was 45°C. The sample injection volume was 5 μl. Stop run MS spectra were collected over an *m/z* range of 140–1500 Da, operating in IDA® mode (information-dependent acquisition). Collision energy was set at 35 (collision energy spread 15). Data processing was carried out using the untargeted data processing program MSDIAL (v. 4.25) with LipidBlast database v. 68.

### Transmission electron microscopy of lipoproteins

CSF samples for transmission electron microscopy (TEM) were concentrated 5 folds using Microcon ultra (Merck) and prepared by negative stain protocol as follows. Five microliters of sample solution was adsorbed onto a 200-mesh nickel grid previously glow discharged using a sputter coater, Leica EM ACE600 (Leica Microsystems, Wetzlar, Germany). After 1 min, excess sample was removed with blotting paper followed by washing on 3 drops of milliQ water. Grid was negatively stained with uranyl acetate 2% in water, blotted again, and air-dried prior observation. Samples were observed using a Talos L120C TEM (Thermo Fisher Scientific, Waltham) operating at 100 κV and images were acquired by Ceta 16M CMOS-based sensor camera. At least three grids were observed for each sample.

### mRNA and protein expression of SOAT2

The human hepatoma cell line HepG2, obtained from the ATCC and human glioblastoma astrocytoma U373-MG cell line, kindly donated by Prof. Zimetti, University of Parma (9) were cultured in DMEM containing 10% FBS, 2 mmol/l l-glutamine, 0.1 U/ml penicillin, and 0.1 μg/ml streptomycin. Normal human astrocytes (NHAs), purchased from Lonza (Walkersville, Maryland), were maintained in astrocyte basal medium supplemented with AGM BulletKit™ (10). All cell lines were cultured at 37°C under a humidified atmosphere containing 5% CO_2_.

For gene expression analysis, confluent U373, NHA, and HepG2 cells were lysed in Trizol Plus reagent (Life Technologies), and RNA was extracted according to the manufacturer's instructions. cDNA was produced by reverse transcription of 0.8 μg of total RNA using the iScript cDNA Synthesis kit (Bio-Rad Laboratories). Amplification of cDNA was carried out with the iTaq Universal SYBR Green Supermix in a MiniOpticon System (Bio-Rad Laboratories, Hercules, CA). SOAT2 and SOAT1 expressions were assessed by quantitative polymerase chain reaction and results normalized on housekeeping expression (β-actin) with ΔΔCt method and compared using HepG2 gene expression to normalize. Primers sequences are listed in the Supplemental Table S1.

For analysis of protein expression, confluent U373, NHA, and HepG2 cells were collected in RIPA buffer (25 mM Tris–HCl, 150 mM NaCl, 1% Igepal, 1% sodium deoxycholate, 0.1% SDS, pH = 8). Cell debris was removed by centrifugation and protein concentration was determined by the microBCA assay (Thermo Fisher Scientific, IL). About 100 μg of extracted protein was digested using S-Trap™ micro spin columns (ProtiFi, Fairport, NY) following a modified protocol (11), replacing TEAB with Tris phosphate in the 2× SDS lysis buffer (10% SDS, 100 mM Tris phosphate, pH 8.5) and binding buffer (90% methanol with 100 mM Tris phosphate, pH 7.55), and using 50 mM ammonium bicarbonate as digestion buffer. Proteins were reduced with 20 mM DTT at 56°C for 45 min, alkylated with 40 mM iodoacetamide at room temperature for 30 min in the dark, acidified with phosphoric acid (final concentration 1.2%), mixed with binding buffer (1:6 v/v), loaded onto S-Trap columns, and digested overnight with trypsin (Sigma-Aldrich) at 37°C. Peptides were sequentially eluted, dried under vacuum (Hetovac, Savant), resuspended in 0.1% formic acid, and quantified using a NanoDrop OneC spectrophotometer (Thermo Fisher Scientific, Wilmington, DE). LC-MS/MS analysis was performed using a Dionex UltiMate 3000 nanoRSLC system (Thermo Fisher Scientific) coupled to a timsTOF mass spectrometer (Bruker Daltonics, Bremen, Germany) operating in data-independent acquisition - parallel accumulation–serial fragmentation mode (12). Trypsin-digested peptides (500–900 ng) were separated on a 50 cm C18 analytical column (Acclaim PepMap RSLC, 75 μm ID, 2 μm particles) at 300 nl/min using a 90-min gradient (4%–98% phase B; 20:80:0.08 H_2_O/acetonitrile/formic acid), ionized via nanoCaptiveSpray™, and analyzed across an *m/z* range of 100–1700 and an ion mobility range of 0.85–1.30 Vs/cm^2^, with 10 data-independent acquisition - parallel accumulation–serial fragmentation cycles (25 Da windows, ∼1.17 s per cycle). Mass accuracy was ensured using an MMI-L Low Concentration Tuning Mix (Agilent Technologies, Santa Clara, CA) and internal calibration with three lock masses (m/z 622.0290, 922.0098, and 1221.9906). Raw data were processed with Bruker Proteomics Suite v.2025c using DiarectDIA + workflows and additionally analyzed using Spectronaut software v19.7 (Biognosys AG, Switzerland), as previously reported (13). Database searches were performed against the UniProt human database (accessed February 2024) using trypsin specificity, carbamidomethylation of cysteine as a fixed modification, oxidation of methionine, and protein N-terminal acetylation as variable modifications, a 1% protein false discovery rate, and protein quantification based on significant peptides.

### Statistical analysis

Analyses were performed in SPSS version 29.0 (IBM Corp., Armonk, NY) and GraphPad Prism 10.4 (GraphPad Software, San Diego, CA). Continuous variables were summarized as mean ± SD if normally distributed, or as median (first and third quartiles) otherwise, and compared between groups using the Student's *t* test or the Wilcoxon rank-sum test, as appropriate. Categorical variables were presented as counts (percentages) and compared using the chi-square test or Fisher's exact test, as appropriate. All tests were two-sided and *P* values < 0.05 were considered statistically significant.

## Results

### CE fatty acid composition in CSF is significantly different from plasma

Plasma and CSF samples were obtained from CN controls without a personal or family history of neurological or psychiatric disorders, recruited in a large study conducted at the IRCCS “San Gerardo dei Tintori” (7). The characteristics of the enrolled control subjects are summarized in Table 1.

**Table 1:** Demographic and clinical data of enrolled subjects

	CN controls	AD
N	4	12
Sex (M/F)	3/1	7/5
Age (years)	72.0 ± 5.1	68.1 ± 5.2
MMSE (score)		19.8 ± 3.7
CDR (score)		1.3 ± 0.7
CSF Disease biomarkers		
Aβ1-42 (pg/ml)	861 (830; 891)	533 (435; 636)
Aβ1-40 (pg/ml)	n.a.	11,592 (9,847; 13,546)
T-tau (pg/ml)	188 (184; 192)	643 (439; 831)
P-tau181 (pg/ml)	34 (24; 44)	100 (74; 133)
Aβ1-42/Aβ1-40	n.a.	0.045 (0.043; 0.051)
Aβ1-42/T-tau	3.21 (2.1; 4.32)	0.74 (0.64; 1.31)
Aβ1-42/P-tau181	21.7 (8.8; 34.6)	4.5 (3.5; 6.7)
Apolipoprotein E genotype (N, %)		
E3/E4	1 (25)	7 (58.3)
E3/E3	3 (75)	4 (33.3)
E2/E3	-	1 (8.3)

Data are reported as mean ± SD or median (first quartile; third quartile), as appropriate.

AD, Alzheimer's disease; CDR, Clinical Dementia Rating Scale; CN, cognitive normal; MMSE, Mini-Mental State Examination.

**Fig. 1:**
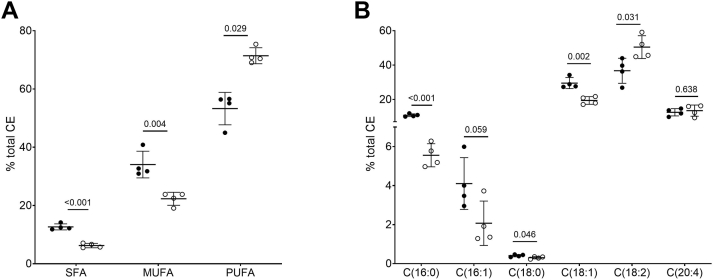
CSF and plasma CE fatty acid (CEFA) composition. Scatter dot plots represent percentage of the specific CE in CSF (black dots) and plasma (white dots) of control subjects (N = 4). A: CSF and plasma CEFA are stratified by saturation degree. B: The most relevant CSF and plasma CEs are shown. Data are expressed as mean ± SD; CSF and plasma were compared by *t* test or Wilcoxon rank-sum test as appropriate. C16:0, palmitate; C16:1, palmitoleate; C18:0, stearate; C18:1, oleate; C18:2, linoleate; C20:4, arachidonate; MUFA, monounsaturated fatty acid; PUFA, polyunsaturated fatty acid; SFA, saturated fatty acid; CSF, cerebrospinal fluid; CE, cholesteryl ester.

The analysis of CSF lipid content revealed the presence of a predominance of esterified cholesterol (61% of total lipid mass), and of similar amounts of phospholipid (19%) and unesterified cholesterol (21%) (Table 2); TGs were not detectable in CSF, in line with their very low levels previously described (14). Cholesteryl ester fatty acid (CEFA) composition in CSF showed that CSF CEs are significantly enriched in saturated fatty acids (SFAs) and monounsaturated fatty acids (MUFAs) and depleted in polyunsaturated fatty acids (PUFAs) compared to plasma CEs (Fig. 1A, Supplemental Table S2). Specifically, palmitate and oleate, typical substrates of the SOAT enzymes (15), were 94% and 52% more abundant in CSF than in plasma, while the major LCAT substrate linoleate was 27% lower in CSF than plasma (Supplemental Table S2, Fig. 1B).

**Table 2:** CSF and plasma lipid/lipoprotein profile of analyzed subjects

	CN controls (n = 4)	AD (n = 12)	*P* value
CSF			
Total cholesterol (mg/dl)	0.460 ± 0.105	0.253 ± 0.057	<0.001
Unesterified cholesterol (mg/dl)	0.159 ± 0.052	0.130 ± 0.031	0.20
Unesterified/total cholesterol	0.356 ± 0.116	0.514 ± 0.073	<0.01
Cholesteryl esters (mg/dl)	0.492 (0.337; 0.685)	0.207 (0.162; 0.231)	<0.01
Phospholipids (mg/dl)	0.152 ± 0.027	0.151 ± 0.037	0.97
Apolipoprotein A-I (mg/dl)	1.06 (0.79; 1.23)	0.82 (0.24; 1.14)	0.22
Apolipoprotein E (mg/dl)	1.69 ± 0.37	2.71 ± 0.59	0.01
CER (nmol/ml/h)	1.30 ± 0.84	0.46 ± 0.44	0.05
Plasma			
Total cholesterol (mg/dl)	164.3 ± 48.4	167.2 ± 39.7	0.91
Unesterified cholesterol (mg/dl)	43.3 ± 12.7	46.6 ± 12.2	0.68
Unesterified/total cholesterol	0.257 ± 0.018	0.283 ± 0.050	0.42
Cholesteryl esters (mg/dl)	203.3 ± 60.7	202.6 ± 52.3	0.98
LDL cholesterol (mg/dl)	88.0 ± 27.4	96.6 ± 35.4	0.70
HDL cholesterol (mg/dl)	50.5 (30.4; 79.7)	55.0 (47.1; 59.8)	0.72
Triglycerides (mg/dl)	113.7 ± 18.2	96.6 ± 35.4	0.44
Phospholipids (mg/dl)	208.0 ± 58.1	184.9 ± 37.2	0.40
Apolipoprotein A-I (mg/dl)	131.0 (118.1; 143.6)	131.0 (118.1; 143.6)	0.83
Apolipoprotein A-II (mg/dl)	28.6 (19.1; 37.8)	27.7 (23.3; 28.7)	0.79
Apolipoprotein E (mg/dl)	2.84 ± 1.09	2.61 ± 0.56	0.70
Apolipoprotein B (mg/dl)	81.8 ± 8.1	85.7 ± 21.7	0.77
LCAT activity (nmol/ml/h)	37.8 ± 5.6	31.6 ± 8.5	0.26
CER (nmol/ml/h)	22.8 ± 7.9	32.9 ± 10.2	0.14

Data are reported as mean ± SD or median (first quartile; third quartile), as appropriate. AD and controls were compared by Student's *t* test or Wilcoxon rank-sum test, as appropriate.

CER, cholesterol esterification rate; CN, cognitively normal.

**Fig. 2:**
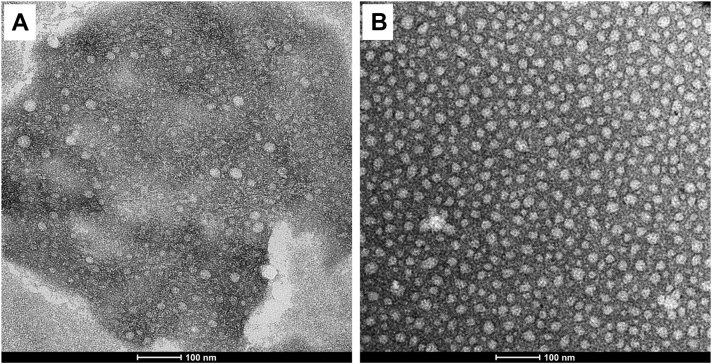
Negative staining transmission electron microscopic (TEM) images of CSF HDL particles. A: CSF HDL particles from a control subject. B: CSF HDL particles from an AD patient. The scale bars represent 100 nm. The images are representative of all independently prepared samples. CSF, cerebrospinal fluid; AD, Alzheimer's disease.

In agreement with the large amount of lipoprotein core lipids (CEs) detected in CSF, analysis of CSF lipoproteins by TEM showed that most particles have spherical shape (Fig. 2A). This result is in line with the absence of discoidal pre-β HDL in CSF, recently reported by our group (7).

### Human astrocytes express SOAT1 but not SOAT2

**Fig. 3:**
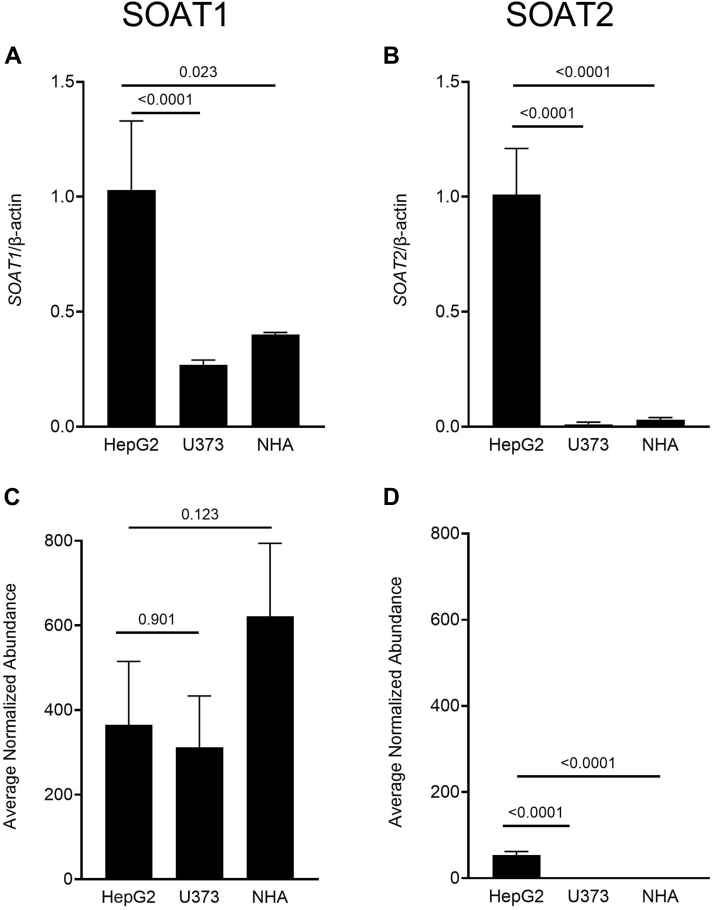
SOAT1 and SOAT2 gene and protein expression in HepG2 cells and astrocytes. SOAT1 gene (A) and protein (C) expression and SOAT2 gene (B) and protein (D) expression in human hepatic cells (HepG2), human astrocytes cell line (U373), and normal human primary astrocytes (NHAs). Data are expressed as mean ± SD; expression in astrocytes was compared to HepG2 expression by *t*-test. SOAT1, sterol O-acyltransferase 1; SOAT2, sterol O-acyltransferase 2.

The CSF CEFA composition let us hypothesize the contribution of SOAT2 in brain lipoprotein CE formation. To investigate the potential presence of SOAT2 in human astrocytes, the lipoprotein-producing cells in the CNS, mRNA, and protein expression were analyzed in U373 and NHA cell lines. Human hepatic cells (HepG2) were used as positive control. As shown in Fig. 3, SOAT1 gene and protein were highly expressed in both U373 and NHA cells, whereas neither SOAT2 gene nor protein was detected in astrocytes.

The CSF CEs are thus likely LCAT-derived despite the different fatty acid composition compared to plasma. The analysis of CSF PL composition indeed revealed that CSF phosphatidylcholine, the major LCAT substrate, was enriched in SFAs and MUFAs (Supplemental Fig. S1).

### CSF CEs are enriched in SFAs in AD patients

As already reported by our group (7), plasma lipids were very similar in AD patients and controls, while CSF lipids were different in the two groups (Table 2). Specifically, in the CSF of AD patients total cholesterol was significantly reduced, the unesterified/total cholesterol ratio was significantly increased, and CEs were significantly reduced, but remaining the major lipid in CSF. In line, lipoproteins are spherical in CSF of AD patients (Fig. 2B). CER, a measure of LCAT activity, was significantly reduced, and apoE significantly increased in the CSF of AD patients (Table 2).

**Fig. 4:**
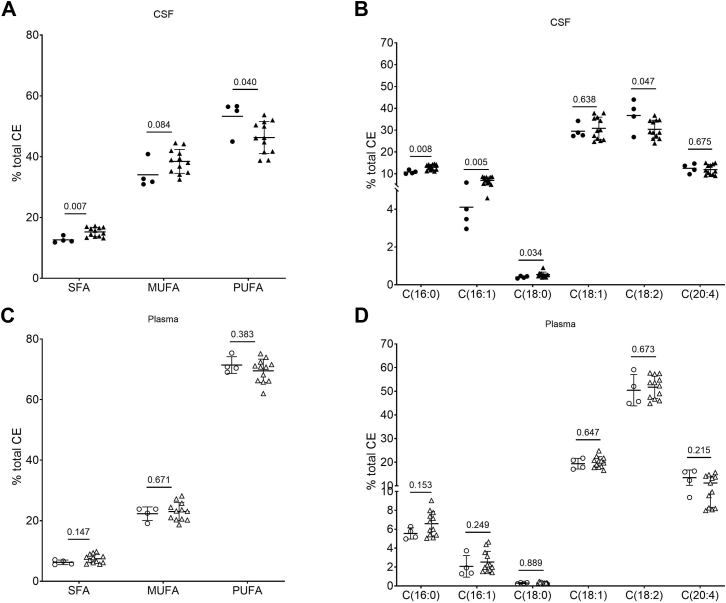
CSF and plasma CE composition in Alzheimer's disease patients and controls subjects. Scatter dot plots represent percentage composition of CEs from CSF (Panels A and B) and plasma (Panels C and D), N = 12 for AD patients and N = 4 for control subjects. Data are expressed as mean ± SD; AD (circles) and controls (triangles) were compared by *t*-test or Wilcoxon rank-sum test as appropriate. *P* < 0.05 is significant. C16:0, palmitate; C16:1, palmitoleate; C18:0, stearate; C18:1, oleate; C18:2, linoleate; C20:4, arachidonate; MUFA, monounsaturated fatty acid; PUFA, polyunsaturated fatty acid; SFA, saturated fatty acid; CE, cholesteryl ester; CSF, cerebrospinal fluid.

Analysis of CEFA composition showed that CSF CEs of AD patients are significantly enriched in SFAs and MUFAs and depleted in PUFAs compared to CSF CEs of control subjects (Fig. 4A, Supplemental Table S2). Specifically, palmitate and oleate were increased by 20% and 4%, respectively, while the major LCAT substrate linoleate was reduced by 17% in CSF from AD patients compared to CSF from controls (Fig. 4B, Supplemental Table S2). No differences were observed in plasma CE composition between controls and AD patients (Fig. 4C, D, Supplemental Table S2).

## Discussion

The present study shows (i) that CSF CEs are enriched in SFAs and MUFAs compared to plasma CEs, and (ii) that CSF CEs are more saturated or monounsaturated in AD patients than in control subjects.

Circulating CEs can be formed in plasma from unesterified cholesterol through the action of the LCAT enzyme or be secreted as such within lipoproteins, being produced by the SOAT2 enzyme. SOAT2 is an intracellular enzyme responsible for cholesterol esterification firstly described (as ACAT2) in liver and intestine of monkeys by the group of Rudel in 1998 (16). Further studies have shown that SOAT2 is present only in hepatocytes and in intestinal mucosal cells (3). Although SOAT1 is located on the cytoplasmic side of the membrane, where it could possibly facilitate CE incorporation into lipid droplets, SOAT2 is predicted to have its active site on the luminal side of the endoplasmic reticulum membrane (17), where lipoprotein assembly and secretion is known to occur. While SOAT2 is highly expressed in mice (3), the enzyme is a minor contributor to circulating CEs in humans as shown by the virtual absence of plasma CEs in carriers of genetic LCAT deficiency (6).

Here, we show that the most common CSF CEs are the SOAT2-derived cholesteryl oleate (C18:1) and cholesteryl palmitate (C16:0), which together account for nearly 40% of CSF CEs, compared to 25% in plasma CEs. On the contrary, cholesteryl linoleate (C18:2), reported to be the enzymatic product of LCAT, is largely the most abundant CE in plasma. The significantly different CEFA composition between CSF and plasma could be explained (i) by the contribution of SOAT2 to CSF CEs or (ii) by a different substrate availability for the LCAT reaction in the CSF. The first hypothesis was ruled out based on the analysis of astrocytes; the lipoprotein-producing cells in the brain, showing that neither *SOAT2* gene nor SOAT2 protein were expressed in these cells. We thus considered the alternative hypothesis, that is, that CSF CEs are LCAT-derived, despite their different fatty acid composition compared to plasma CEs. LCAT catalyzes the transacylation of the sn-2 fatty acid of lecithin, which in plasma is typically polyunsaturated, to the free 3-OH group of cholesterol, generating CE and lysolecithin. Analysis of the fatty acid composition of phosphatidylcholine in plasma and CSF showed that oleic acid (C18:1) was by far the most abundant sn-2 fatty acid of CSF phospholipids, while, as expected, linoleic acid (C18:2) was the most common fatty acid in plasma. These results suggest that LCAT can well use MUFA when available in sn-2 of phosphatidylcholine. An alternative potential explanation for the difference in CEFA composition between plasma and CSF could be the different LCAT activator; indeed, apoA-I is the major LCAT activator in plasma, while apoE activates LCAT in the CSF. ApoA-I is certainly more potent than apoE in activating the LCAT enzyme (18); however, the activator does unlikely direct the enzyme substrate specificity, as shown by the similar CEFA composition in plasma HDL and VLDL (19), where apoA-I or apoE is the major LCAT activator, respectively.

In the second part of the present study, we extended our previous evaluation of cholesterol esterification in AD patients (7) by analyzing the CEFA composition in CSF and plasma of AD patients and control subjects. Interestingly, the results show that CSF CEs are enriched in SFAs and MUFAs in AD patients compared to control subjects, with a specific increase in palmitate (C16:0) and oleate (C18:1). Thus, CSF of AD patients not only contains less CEs, likely because of a reduced LCAT activity (7), but CE composition is also altered. A role of the *APOE* genotype in the observed results is unlikely since the apoE4 isoform, prevalent in AD patients and infrequent in control subjects, does activate LCAT equally to apoE3 isoform (18), the most common in controls. Our observation is consistent with a previous report showing that oleic acid accumulates in hippocampus of animal models of AD (20), although the cited study evaluated only TGs fatty acid composition and not CEs or phospholipids. The increase in MUFAs in the brains of AD animal models was attributed to enhanced activity of fatty acid desaturases (20), and notably infusion of a desaturase inhibitor ameliorated cognitive function in an AD animal model (21). Whether desaturase inhibition would also correct altered CE fatty acid composition in CSF of AD patients remains to be investigated.

In conclusion, the present data support the central role of LCAT in the formation of CEs in the CSF and confirm that the cholesterol esterification pathway is hampered in the CSF of AD patients. These results suggest that LCAT-mediated esterification could represent a novel pharmacological target in AD.

Two major limitations of our study should be acknowledged. First, the number of analyzed AD patients and control subjects was small, primarily due to the restricted availability of CSF samples from control subjects. Second, this study does not establish a causal link between altered CSF CEs and disease severity or progression in AD, which warrants further investigation.

## Data Availability

The data that support the findings of this study are available from the corresponding author on realistic request.

## Supplemental Data

This article contains supplemental data.

## Conflict of Interest

The authors declare that they have no conflicts of interest with the contents of this article.
