# Supplementary material for: Cerebrospinal fluid CEFA composition is enriched in saturated fatty acids and it is altered in Alzheimer’s disease
Source: J Lipid Res. 2026 Apr 3;67(5):101034. doi: 10.1016/j.jlr.2026.101034 (PMC13137020; doi:10.1016/j.jlr.2026.101034)
Supplement: Supplemental Tables S1 and S2 [file mmc1.docx]

**Cerebrospinal fluid CEFA composition is enriched in saturated fatty acids and it is altered in Alzheimer’s Disease**

**Authors:** Chiara Pavanello, Alice Ossoli, Chiara Comi, Marta Turri, Lucio Tremolizzo, Elisa Conti, Paolo D’Incecco, Alberto Barbiroli, Paolo Parini, Nico Mitro, Donatella Caruso, Giulia Sierri, Francesca Re, Clizia Chinello, Claudia Fumagalli, Fulvio Magni, Karl Fernandes, Laura Calabresi

**Supplemental Table S1. List of primers for RT-PCR on HepG2, U373 and NHA**

| Gene | Fwd | Rev |
| --- | --- | --- |
| Human SOAT1 | GTG CGC TCT CAC AAC CTT TTC | GGT GCT CTC AAA TCC TTC GCT |
| Human SOAT2 | CAT GTT CAT CGC TGG CCT G | CAG CCT GCC CTC ATC AAT G |
| Human β-actin | CTG GAC TTC GAG CAA GAG ATG | CCA TGC CCA GGA AGG AGG |

**Supplemental Table S2. CSF and plasma cholesteryl ester (CE) distribution in CN controls and AD patients**

|  | CSF | | | Plasma | | |  | | |
| --- | --- | --- | --- | --- | --- | --- | --- | --- | --- |
| Cholesteryl esters | Control subjects | AD | *P*  *CN vs AD* | Control subjects | AD | *P*  *Controls vs AD* | | *P*  *CSF vs Plasma (Controls)* | *P*  *CSF vs Plasma (AD)* |
| C 14:0 (%) | 0.64 (0.56; 0.78) | 1.14 (0.92; 1.34) | 0.006 | 0.22 (0.13; 0.29) | 0.23 (0.20; 0.37) | 0.431 | | < 0.001 | <0.001 |
| C 15:0 (%) | 0.51±0.11 | 0.75±0.14 | 0.027 | 0.09 (0.06; 0.11) | 0.11 (0.09; 0.13) | 0.363 | | < 0.001 | <0.001 |
| C 16:0 (%) | 10.80±0.82 | 12.98±1.31 | 0.008 | 5.56±0.59 | 6.59±1.30 | 0.153 | | < 0.001 | <0.001 |
| C 17:0 (%) | 0.27 (0.22; 0.33) | 0.27 (0.24; 0.40) | 0.585 | 0.10±0.03 | 0.13±0.04 | 0.153 | | 0.001 | <0.001 |
| C 18:0 (%) | 0.42 (0.35; 0.47) | 0.53 (0.46; 0.59) | 0.034 | 0.30±0.06 | 0.31±0.08 | 0.889 | | 0.046 | <0.001 |
| C 16:1 (%) | 4.11±1.33 | 6.98±1.54 | 0.005 | 1.64 (1.31; 3.27) | 2.11 (1.57; 3.45) | 0.249 | | 0.059 | <0.001 |
| C 17:1 (%) | 0.44±0.14 | 0.63±0.11 | 0.009 | 0.14 (0.11; 0.25) | 0.20 (0.16; 0.27) | 0.163 | | 0.014 | <0.001 |
| C 18:1 (%) | 29.52±3.21 | 30.84±5.11 | 0.638 | 19.39±2.25 | 20.02±2.32 | 0.647 | | 0.002 | <0.001 |
| C 20:1 (%) | 0.0 (0.0; 0.0) | 0.0 (0.0; 0.0) | n.a | 0.46±0.16 | 0.41±0.16 | 0.597 | | 0.014 | <0.001 |
| C 22:1 (%) | 0.0 (0.0; 0.0) | 0.0 (0.0; 0.0) | n.a | 0.23±0.03 | 0.23±0.04 | 0.801 | | 0.014 | <0.001 |
| C 18:2 (%) | 36.70±7.29 | 30.38±4.20 | 0.047 | 50.45±6.63 | 51.8±4.75 | 0.673 | | 0.031 | <0.001 |
| C 20:2 (%) | 0.0 (0.0; 0.0) | 0.0 (0.0; 0.0) | n.a | 1.26 (1.15; 1.49) | 1.17 (0.87; 1.63) | 0.905 | | 0.014 | <0.001 |
| C 22:2 (%) | 0.0 (0.0; 0.0) | 0.0 (0.0; 0.0) | n.a | 0.68 (0.61; 0.80) | 0.77 (0.75; 0.78) | 0.286 | | 0.014 | <0.001 |
| C 18:3 (%) | 1.00±0.23 | 0.92±0.20 | 0.508 | 1.51 (1.25; 2.45) | 1.76 (1.42; 2.37) | 0.671 | | 0.095 | <0.001 |
| C 20:3 (%) | 1.07±0.28 | 1.39±0.59 | 0.329 | 1.27±0.32 | 1.18±0.36 | 0.642 | | 0.381 | 0.300 |
| C 20:4 (%) | 12.51±2.14 | 11.95±2.33 | 0.675 | 13.48±3.26 | 11.23±2.92 | 0.215 | | 0.638 | 0.436 |
| C 22:4 (%) | 0.0 (0.0; 0.0) | 0.0 (0.0; 0.0) | n.a. | 0.47 (0.35; 0.67) | 0.26 (0.23; 0.67) | 0.413 | | 0.014 | <0.001 |
| C 24:4 (%) | 0.0 (0.0; 0.0) | 0.0 (0.0; 0.0) | n.a | 0.19±0.02 | 0.18±0.06 | 0.657 | | 0.029 | <0.001 |
| C 20:5 (%) | 0.81±0.33 | 0.57±0.32 | 0.213 | 0.77 (0.56; 1.31) | 0.95 (0.75; 1.56) | 0.363 | | 0.799 | 0.001 |
| C 22:6 (%) | 1.17±0.16 | 1.09±0.17 | 0.403 | 0.98 (0.63; 1.15) | 0.90 (0.79; 1.05) | 0.952 | | 0.164 | 0.061 |
| Total SFA (%) | 12.67±1.04 | 15.3±1.50 | 0.007 | 6.26±0.76 | 7.44±1.46 | 0.147 | | < 0.001 | <0.001 |
| Total MUFA(%) | 34.07±4.58 | 38.5±3.93 | 0.084 | 22.32±2.24 | 23.03±2.98 | 0.671 | | 0.004 | <0.001 |
| Total PUFA (%) | 53.28±5.57 | 46.29±5.25 | 0.040 | 71.42±2.75 | 69.53±3.86 | 0.383 | | 0.029 | <0.001 |

AD, Alzheimer’s disease; MUFA, monounsaturated fatty acids; n.a., not applicable; PUFA, polyunsaturated fatty acids; SFA, saturated fatty acids. Data are reported as mean ± SD or median (1^st^; 3^rd^ quartile), as appropriate. *P* are for Wilcoxon rank-sum test or Student t-test, as appropriate.

**
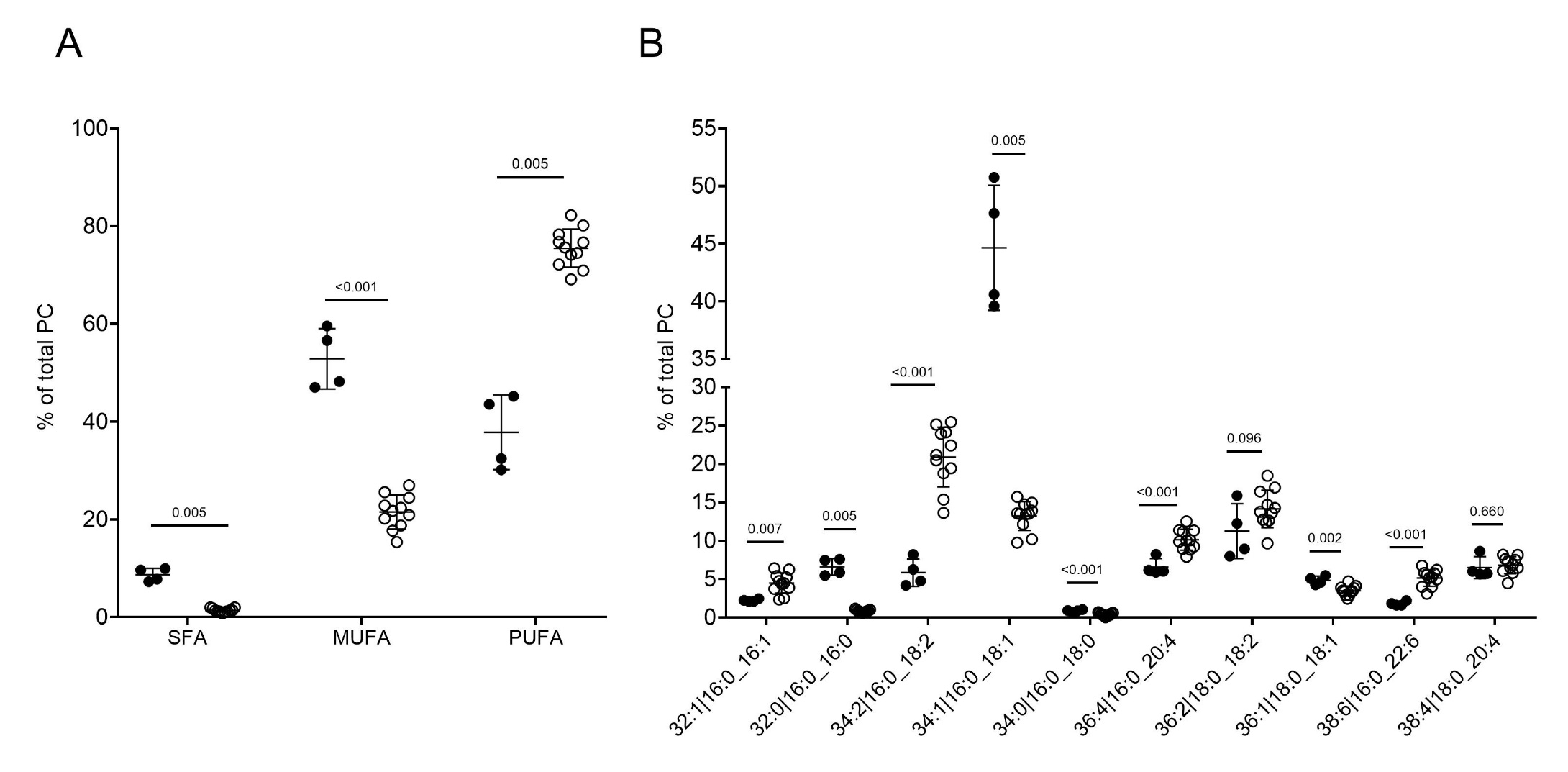
Fig. S1. CSF and plasma phosphatidylcholine fatty acid composition**. Scatter dot plots represent percentage of individual PC fatty acid species in CSF (black dots) and plasma (white dots) of control subjects (N=4). (A) CSF and plasma phosphatidylcholine FA are stratified by saturation degree. (B) The most abundant PC species in each compartment are shown. Data are expressed as mean±SD; CSF and plasma were compared by t-test or Wilcoxon rank-sum as appropriate; MUFA, monounsaturated fatty acids; PUFA, polyunsaturated fatty acids; SFA, saturated fatty acids.
